# Role of secreted frizzled-related protein 4 in prediabetes and type 2 diabetes: a cross sectional study

**DOI:** 10.1186/s12902-024-01613-5

**Published:** 2024-06-04

**Authors:** Katy Sánchez-Pozos, MA Granados-Silvestre, NG Nieto-Velázquez, María Alicia Mejía-Blanquel, Natsyelli Galicia-Martínez, Jessica Mandujano-Cerón, Joel Jaimes-Santoyo, María Guadalupe Ortiz-López

**Affiliations:** Research Division, Hospital Juárez de México, Av Instituto Politécnico Nacional 5160, Magdalena de las Salinas, Gustavo A. Madero, Mexico City, 07760 México

**Keywords:** Type 2 diabetes, Prediabetes, Biomarker, SFRP4; insulin resistance

## Abstract

**Background:**

Type 2 diabetes (T2D) has become an epidemic. Delays in diagnosis and as a consequent late treatment has resulted in high prevalence of complications and mortality. Secreted frizzled-related protein 4 (SFRP4), has been recently identified as a potential early biomarker of T2D related to obesity, due to its association with low grade inflammation in adipose tissue and impaired glucose metabolism. We aimed to evaluate the role of SFRP4 in prediabetes and T2D in a Mexican population.

**Methods:**

This was a cross-sectional study that included 80 subjects with T2D, 50 subjects with prediabetes and 50 healthy individuals. Fasting SFRP4 and insulin concentrations were measured by ELISA. Human serum IL-10, IL-6, IL-1β and IL-8 levels were quantified by flow cytometry. Genotyping was performed by TaqMan® probes.

**Results:**

Prediabetes and T2D patients had significantly higher SFRP4 levels than controls (*P* < 0.05). In turn, prediabetes subjects had higher SFRP4 concentrations than control subjects (*P* < 0.05). Additionally, the prediabetes and T2D groups had higher concentrations of proinflammatory molecules such as IL-6, IL-1β and IL-8, and lower concentrations of IL-10, an anti-inflammatory cytokine, than controls (*P* < 0.001). The serum SFRP4 concentrations were positively correlated with parameters that are elevated in prediabetes and T2D states, such as, HbA1c and homeostasis model assessment insulin resistance (HOMA-IR), (*r* = 0.168 and 0.248, respectively, *P* < 0.05). Also, serum SFRP4 concentrations were positively correlated with concentrations of pro-inflammatory molecules (CRP, IL-6, IL-1β and IL-8) and negatively correlated with the anti-inflammatory molecule IL-10, even after adjusting for body mass index and age (*P* < 0.001). The genetic variant rs4720265 was correlated with low HDL concentrations in T2D (*P* < 0.05).

**Conclusions:**

SFRP4 correlates positively with the stage of prediabetes, suggesting that it may be an early biomarker to predict the risk of developing diabetes in people with high serum concentrations of SFRP4, although further longitudinal studies are required.

## Introduction

Diabetes and cardiovascular disease are leading causes of disability and death worldwide. According to the International Diabetes Federation (IDF), the incidence of diabetes, specifically type 2 diabetes (T2D), will rise from 536.6 million in 2021 to 783.2 million by 2045 [1]. The need for new biomarkers for the early diagnosis of diabetes in people at risk is therefore a major challenge for contemporary laboratory medicine.

In Mexico, diabetes is the second leading cause of death and the leading cause of years of healthy life lost; for these reasons, in 2016, diabetes was declared an epidemiological emergency in Mexico by the National Health System [2]. It is well known that the defects in glucose metabolism that trigger T2D begin many years before the diagnosis of the disease [3, 4]. One of the main risk factors for T2D development is prediabetes, which manifests as impaired glucose tolerance (IGT) (2-h plasmatic glucose levels during a 75-g oral glucose tolerance test [OGTT] from 140 to 199 mg/dL) and/or impaired fasting glucose (100–125 mg/dL). Additionally, prediabetes is associated with obesity, particularly abdominal or visceral obesity [5, 6]. In Mexico, the prevalence of prediabetes is approximately 22.1%, of which approximately 5–10% will develop T2D, according to the projections indicated by Nathan et al., and Forouhi et al. [7–9]. Hence, diagnostic targets that allow us to identify the population at risk are especially useful for taking preventive measures. In this context, for prediabetic individuals, lifestyle modification is crucial to prevent diabetes development, with a 40–70% relative risk reduction [4].

Secreted frizzled-related protein 4 (SFRP4) is a member of the SFRP family that contains a cysteine-rich domain homologous to the putative Wnt-binding site of frizzled proteins [10]. The SFRP4 protein contains 346 amino acids with a weight of 39.9 kDa [11]. The protein encoded by the *SFRP4* gene is an extracellular regulator of the Wnt pathway and has roles in tissue development, cancer and phosphate metabolism [10].

SFRP4 is expressed in the endometrial stroma, ovaries, pancreas, stomach, colon, lung, skeletal muscle, liver, and the eye, among other tissues. In addition, receptors for SFRP4 have been found in many organs, including pancreatic β cells. SFRP4 therefore controls a multitude of biological functions in humans [10, 12].

Impaired SFRP4 levels are involved in different pathologies, especially metabolic disorders such as obesity and diabetes [13]. In this sense, individuals with increased levels of SFRP4 in the blood are five times more likely to develop diabetes in the coming years [14]. Moreover, the association of SFRP4 with T2D and metabolic syndrome components has been observed in several studies. SFRP4 has been associated with a larger waist circumference, increased body mass index (BMI) and increased triglyceride levels [15]. Additionally, SFRP4 expression is upregulated in obese individuals and is correlated with insulin resistance [16].

Mahdi et al. [14] suggested that SFRP4 acts by decreasing insulin secretion from pancreatic beta cells, preventing the transcription of angiogenesis-associated genes, including vascular endothelial growth factor (VEGF), and modulating Wnt signaling (a pathway involved in glucose metabolism). SFRP4 has been described as involved in the metabolism of glucose and lipids by interacting with Wnt ligands. Elevated levels of SFRP4 therefore caused reduced glucose tolerance through decreased islet expression of Ca (2+) channels and suppressed insulin exocytosis. In the same study, the authors showed that SFRP4 is overexpressed in the islets of patients with T2D. Another study demonstrated that serum SFRP4 levels are inversely correlated with the first phase of glucose-stimulated insulin secretion in individuals with different glucose tolerances [17]. Furthermore, SFRP4 was associated with inflammatory markers. In this sense, interleukin-1β has been reported to cause the secretion of SFRP4 from pancreatic islets [14].

Only one study has explored the genetic contribution of SFRP4 to metabolic disorders through the identification of genetic variants in body composition. Boudin et al. [18] found six genetic variants or SNPs (single nucleotide polymorphisms) associated with body mineral density, hip geometry parameters, height and percentage body fat. The authors concluded that genetic variation contributes to hip fracture risk, percentage body fat and height in a Danish male population. We therefore hypothesize that genetic variation in SFRP4 could be related to metabolic disorders, specifically obesity.

As diabetes is a disease that does not result in symptoms in its early stages, it is necessary to identify potential biomarkers that can distinguish subjects at risk to develop T2D to implement preventive measures. Thus, the aim of the present study was to evaluate SFRP4 in patients with prediabetes and T2D, as well as its relationship with inflammatory markers (interleukins), to elucidate the role of SFRP4 in insulin resistance as prelude of T2D.

## Methods

### Subjects

This is a cross-sectional study that included a total of 180 volunteers who attended Hospital Juarez de Mexico, a tertiary care hospital. The subjects were divided into three groups according to their glycated hemoglobin (HbA1c) level: the Control group (*n* = 50) with a Hb1Ac of less than 5.7%; the Prediabetes group (*n* = 50) with Hb1Ac between 5.7% and 6.4%; and T2D (*n* = 80) with Hb1Ac ≥ 6.5%. The classification of the groups was based on the diagnostic criteria of the American Diabetes Association 2022 [5].

Patients with anemia, type 1 diabetes, gestational diabetes, uncontrolled hypertension, active cancer, heart failure, liver or kidney disease, autoimmune diseases, cotreatment with corticosteroids or estrogens, conditions that can cause hyperglycemia, addiction to alcohol or illegal drugs, dementia or severe psychiatric disorders were not included in this study.

### Anthropometric parameters

The following anthropometric parameters were recorded for all participants according to standard protocols: body weight, height, and waist (WC) and hip circumference. The same technicians took all measures. Body mass index (BMI) was calculated with the formula height/weight^2^ (Kg/m^2^). The waist-to-hip ratio (WHR) was also calculated with the waist circumference/hip circumference formula. Blood pressure was measured twice with a standard mercury manometer.

### Biochemical parameters

Measurements of glucose, triglycerides, cholesterol, high density lipoprotein cholesterol (HDL-C), low density lipoprotein cholesterol (LDL-C), very low-density lipoprotein cholesterol (VLDL-C), creatinine, blood urea nitrogen, uric acid, alanine transferase (ALT) and aspartate aminotransferase (AST) levels were performed at the Hospital Juárez de México with the ADVIA 2000 by commercially available standardized methods according to the manufacturer’s instructions. Analytical quality determinations were monitored using an internal quality control system and the participation of an external quality assurance program. HbA1c was measured in total blood on an ADVIA 2120i Hematology System Siemens Analyzer. PCR levels in peripheral blood samples were quantified by nephelometry with intensifying particles (Siemens CardioPhase® hsCRP, Marburg, Germany) using the BNII system, according to the manufacturer’s manual.

Insulin resistance was estimated by the homeostasis model assessment insulin resistance (HOMA-IR), was calculated by the formula [19, 20]:

$$HOMA-IR=\frac{fasting insulin \left(\frac{mU}{mL}\right)\times fasting plasma glucose \left(\frac{mg}{dL}\right)}{405}$$

The homeostasis model assessment of β-cell function (HOMA-β), was assessed by the following formula [20]:

$$HOMA-\beta =\frac{360\times fasting insulin \left(\frac{mU}{mL}\right)}{fasting plasma glucose \left(\frac{mg}{dL}\right)-63}$$

### Assessment of serum SFRP4 concentrations

Serum SFRP4 levels were determined by enzyme-linked immunosorbent assays (ELISA) according to the manufacturer´s instructions (ELISA KIT, Secreted Frizzled Related Protein 4 (SFRP4), Mybiosource**®**, catalog number MBS2020567). Control positives were included in the assay. According to the manufacturer’s instructions, the sensitivity of the kit is defined as a minimum detectable dose of SFRP4 of less than 25.8 pg/mL. The specificity was determined with cross-reactivity; no significant cross-reactivity or interference was observed.

### Flow cytometry

Human serum interleukin-10 (IL-10), interleukin-6 (IL-6), interleukin-1 beta (IL-1β) and interleukin-8 (IL-8) levels were quantified using bead-based assays following the same principle as a sandwich immunoassay (BD Cytometric Bead Array; BD Biosciences, San Jose, CA, USA) in accordance with the manufacturer’s instructions. Fluorescence from the beads was detected using a BD Accuri C6 flow cytometer system (Becton Dickinson) and analyzed with the FCAP Array V3.0 software.

### Genotyping

We included 184 T2D patients in addition to the 80 T2D patients for the genotypic analysis, resulting in a total of 264 T2D patients, in order to increase the statistical power in the analysis. A sample of peripheral blood was collected in EDTA tubes. Genomic DNA was extracted from the blood sample according per Miller et al. [21]. The obtained DNA was quantified by a Nanodrop®, and the integrity was confirmed by 1% agarose electrophoresis. Genotyping was carried out by qPCR through the TaqMan assay (Thermo Fisher Scientific, USA): rs4720265 (C__27961703_10). Software CFX Manager Bio-Rad version 2.1 was used to perform allelic discrimination. Genotyping accuracy was assessed by the inclusion of sample duplicates and negative controls. Call rates exceeded 95%, and no discordant genotypes were observed.

### Statistical analysis

To determine the sample size of the present study, the formula for calculating proportions in infinite populations was used. Therefore, a sample size of at least 180 participants was determined taking into account the following assumptions: prevalence of people with prediabetes in Mexico 22%; a statistical power of 80%; a precision of 6% and a confidence level of 95% [7].

The statistical analyses were performed using the statistical software package SPSS 21.0 (SPPSS Inc., Chicago Illinois). The results are presented as the mean ± standard deviation (SD) or as the median (25th–75th percentiles) according to data normality. Depending on the data distribution, ANOVA or the Kruskal‒Wallis test were used for group comparisons. Interrelationships between variables were analyzed by Spearman correlation analysis and partial correlation analysis. P values < 0.05 (two tailed) were reported as statistically significant.

## Results

### Clinical and biochemical characteristics of participants

**Fig. 1:**
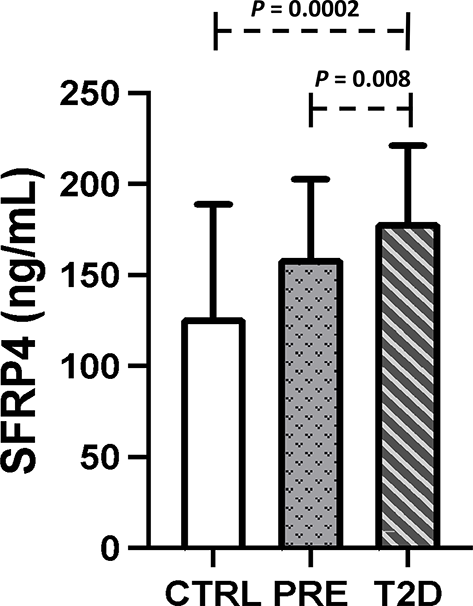
Serum SFRP4 levels in the three study groups. Columns represent the mean with standard deviation. CTRL, control group; PRE, Prediabetes group; T2D, type 2 diabetes group. The Kruskal-Wallis test was performed

The participants were classified according to their diabetes status into control, prediabetes and T2D groups (Table 1). As it can be seen the median of T2D duration was one year. In this context 45% of the patients were recently diagnosed with T2D, therefore these patients did not have treatment at the moment of their inclusion in the study. In addition, 47.5% of patients were receiving metformin. The comparisons among groups showed significant differences in serum glucose and HbA1c, and the T2D group had the highest values. Additionally, the T2D group had higher triglyceride and VLDL-C levels than the control group. The T2D group had the highest levels of SFRP4 compared with the prediabetes and control groups (Table 1; Fig. 1). The concentrations of proinflammatory molecules such as IL-6, IL-1β and IL-8 were increased in the T2D and prediabetes groups compared with the control group. In contrast, the levels of IL-10, an anti-inflammatory molecule, were diminished in the prediabetes and T2D groups.

**Table 1:** Clinical and biochemical characteristics of participants

Characteristic			Control	Prediabetes	T2D	*P*	Total	
N (M/F)			50 (33/16)	50 (23/27)	80 (46/34)		Min	Max
Age (years)			55.9 ± 14.2	53.4 ± 9.8	55.2 ± 8.3	0.483	29	85
Duration of T2D (years)			----	----	**1.0 (0, 5.0)**	---	0	21
BMI (kg/m^2^)			28.9 ± 5.8^b,c^	31.4 ± 5.5^a^	31.3 ± 4.9^a^	0.029	19.6	48.1
WC (cm)		F	94.6 ± 14.1^c^	103.3 ± 14.8	103.0 ± 13.5^a^	0.029	72	147
		M	95.9 ± 7.2	105.5 ± 14.9	101.4 ± 11.4	0.052		
WHR		F	0.91 (0.89, 0.94)^c^	0.93 (0.90, 0.97)	0.95 (0.91, 0.98)^a^	0.028	0.59	1.18
		M	0.98 (0.94, 1.00)	0.97 (0.95, 1.02)	0.98 (0.94, 1.01)	0.987		
SBP (mmHg)			122 (119, 137)	129 (118, 138)	130 (120, 148)	0.439	80	182
DBP (mmHg)			80 (77, 89)	81 (71, 90)	80 (71, 90)	0.564	50	130
Glucose (mg/dL)			85 (80, 94)^b,c^	100 (90, 106)^a,c^	133 (102, 167)^a,b^	7.9 × 10^− 14^	60	383
Insulin (mIU/L)			7.4 (3.6, 10.1)^b, c^	9.7 (7.1, 14.9)^a^	9.1 (6.1, 14.9)^a^	0.003	2.0	177.0
HbA1c (%)			5.6 (5.4, 5.7)^b, c^	6.1 (5.8, 6.2)^a, c^	7.3 (6.5, 8.5)^a, b^	< 2.2 × 10^− 16^	4.4	17.1
HOMA-IR			1.6 (0.7, 2.5)^b, c^	2.6 (1.8, 3.5)^a^	3.3 (1.7, 5.9)^a^	3.5 × 10^− 6^	0.28	39.3
HOMA-B			89.7 (63.6, 153.2)^c^	95.5 (71.8, 150.0)^c^	44.8 (26.8, 87.5)^a, b^		7.29	2360.0
Triglycerides (mg/dL)			158.4 ± 73.1^c^	182.3 ± 100.4	222.9 ± 180.3^a^	0.031	31	790
Hypertriglyceridemia (%)			46.9^c^	53.2^c^	70.5^a, b^	0.013	---	---
Total Cholesterol (mg/dL)			183.4 ± 33.1	183.9 ± 32.6	192.9 ± 42.3	0.272	102	287
HDL-C (mg/dL)	F		49.1 ± 13.8	45.8 ± 9.4	49.0 ± 9.2	0.446	25.8	83.1
	M		41.7 ± 9.9	42.4 ± 9.1	39.7 ± 9.9	0.549		
Low HDL-C (%)			63.3^b^	78.7^a^	74.7	0.019	---	---
LDL-C (mg/dL)			117.0 ± 33.9	119.3 ± 37.8	125.2 ± 40.2	0.445	30.0	225.7
VLDL-C (mg/dL)			29.0 (22.7, 36.0)^c^	33.0 (24.5, 42.5)	36.0 (29.0, 55.0)^a^	0.007	10	288
Creatinine (mg/dL)	F		0.68 (0.64, 0.81)	0.70 (0.63, 0.80)	0.67 (0.58, 0.84)	0.730	0.46	1.35
	M		0.82 (0.73, 0.97)	0.93 (0.80, 0.97)	0.93 (0.86, 1.00)	0.051		
BUN (mg/dL)			14 (12, 17)	15 (12, 17)	15 (13, 17)	0.408	8	37
Urea (mg/dL)			30 (26, 36)	32 (27, 36)	32 (28, 36)	0.514	17	62
AST (U/L)			24 (21, 26)	25 (21, 34)	28 (23, 34)	0.121	10	107
ALT (U/L)			27 (21, 34)	31 (23, 48)	34 (22, 44)	0.068	4	167
SFRP4 (ng/mL)			144.9 (60.4, 183.9)^c^	166.3 (142.1-195.1)^c^	189.7 (168.2-212.6)^a, b^	6.2 × 10^− 7^	16.5	218.6
CPR (mg/L)			0.31 (0.09, 0.59)	0.37 (0.21, 0.74)	0.45 (0.15, 0.81)	0.331	0.02	2.98
IL-6 (pg/mL)			6.5 (4.6, 8.2)^b, c^	32.9 (8.9, 35.7)^a^	33.9 (30.5, 35.7)^a^	< 2.2 × 10^− 16^	3.39	66.7
IL-1β (pg/mL)			5.7 (4.8, 6.4)^b, c^	31.7 (8.5, 34.1)^a^	32.2 (27.6, 34.1)^a^	< 2.2 × 10^− 16^	3.7	78.7
IL-8 (pg/mL)			14.0 (11.4, 15.5)^b, c^	26.8 (17.1, 29.9)^a^	29.6 (26.8, 30.9)^a^	< 2.2 × 10^− 16^	9.3	66.5
IL-10 (pg/mL)			56.0 (54.0, 64.2)^b, c^	35.6 (33.0, 53.0)^a^	35.3 (33.0, 37.7)^a^	< 2.2 × 10^− 16^	21.1	69.4

Data are presented as the means ± SDs or as medians (25th -75th percentiles). BMI, body mass index; WC, waist circumference; WHR, waist hip ratio; SBP, systolic blood pressure; DBP, diastolic blood pressure; HbA1c, glycated hemoglobin; HOMA-IR, homeostasis model assessment insulin resistance; HDL-C, high-density lipoprotein; LDL-C, low-density lipoprotein; VLDL-C, very low-density lipoprotein; BUN, blood urea nitrogen; AST, aspartate transaminase; ALT, alanine transaminase; SFRP4, secreted frizzled-related protein 4; CPR, C-protein reactive; Il-6, interleukin 6; IL-1b, interleukin 1 beta; IL-8, interleukin 8; IL-10, interleukin 10. ^a^*P* < 0.05 vs. control; ^b^*P* < 0.05 vs. Prediabetes and ^c^*P* < 0.05 vs. T2D

### Serum interleukin concentrations

In Table 1, the serum concentrations of proinflammatory and anti-inflammatory factors are shown. There were no differences in CRP levels among the studied groups. However, the levels of the proinflammatory interleukins IL-6, IL-1b and IL-8 were increased in the T2D and prediabetes groups compared with the control group. However, serum concentrations of IL-10 were reduced in T2D and prediabetes patients compared with controls.

### Relationship between serum SFRP4 concentrations and anthropometric and biochemical parameters

**Fig. 2:**
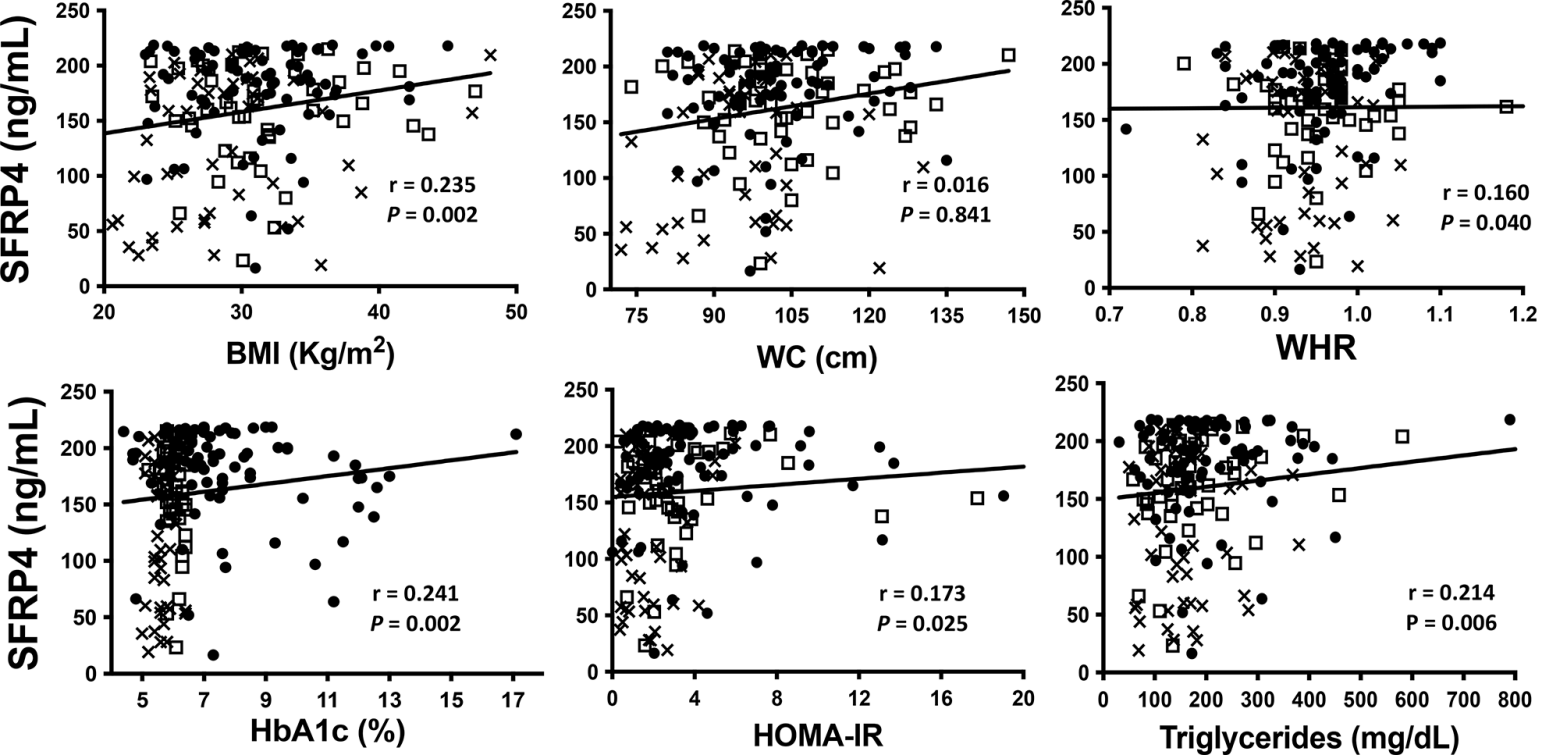
Spearman correlations between SFRP4 and anthropometric and biochemical parameters adjusted by BMI, age and sex. *P* < 0.05 was considered significant. Abbreviations: BMI, body mass index; WC, waist circumference; WHR, waist hip ratio; HbA1c, glycated hemoglobin; HOMA-IR, homeostasis model assessment insulin resistance. Crosses represent control group; white squares represent prediabetes group and black circles represent T2D group

The correlation analysis showed that serum SFRP4 concentrations were positively correlated with BMI, WC, WHR, HbA1c, triglycerides and HOMA-IR (*r* = 0.161, 0.156, 0.204, 0.260, 0.193 and 0.209, respectively, *P* < 0.05). As shown in Fig. 2, after adjustment for BMI, age and sex, the correlations between SFRP4 levels and WC and WHR were lost. Nonetheless, the correlation with BMI, HbA1c, HOMA-IR and triglycerides remained (*r* = 0.235, 0.241, 0.173 and 0.214, respectively, *P* < 0.05).

### Relationship between serum SFRP4 concentrations and serum interleukin concentrations

**Fig. 3:**
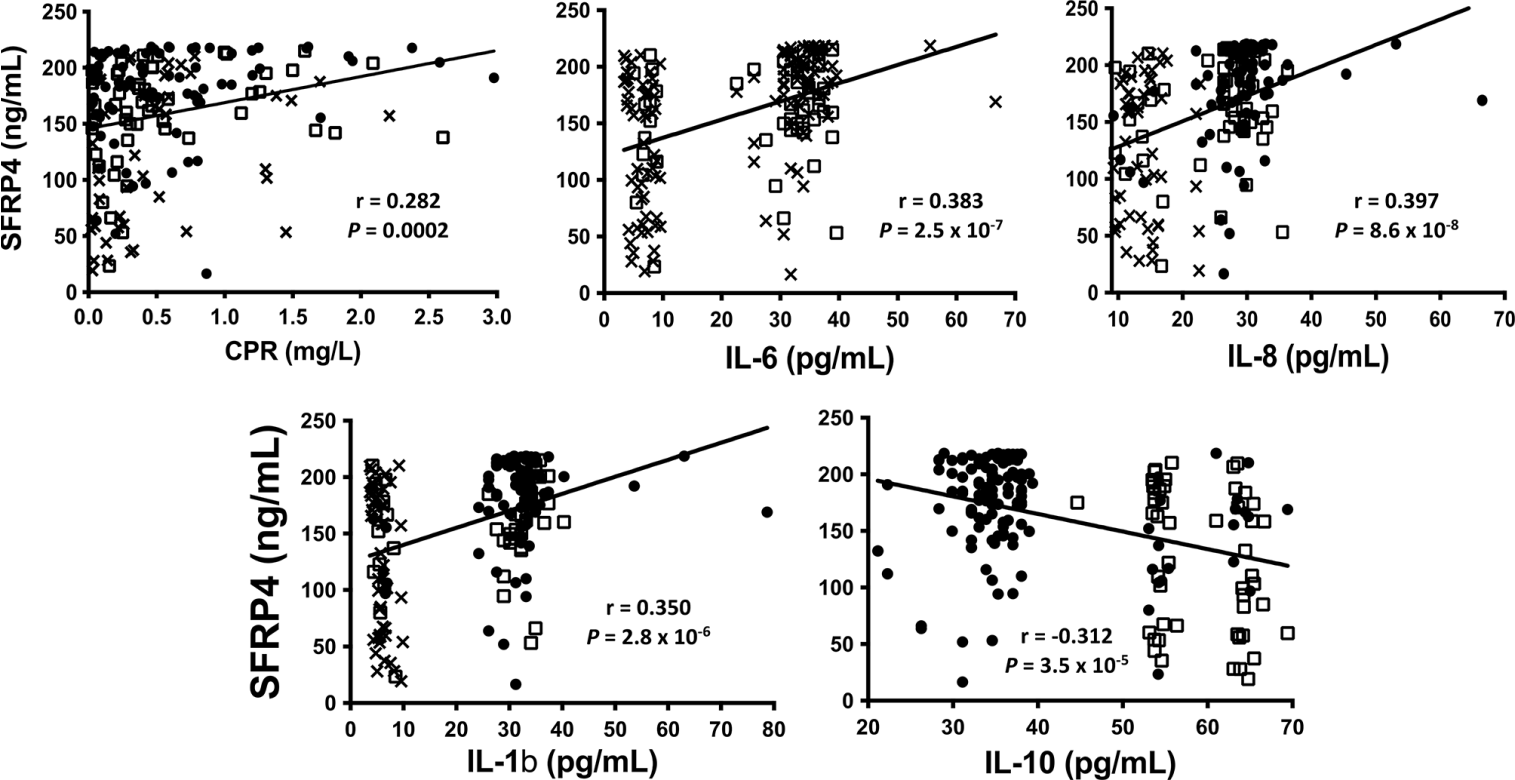
Spearman correlations between SFRP4 and pro-inflammatory molecules. *P* < 0.05 was considered significant Correlations were adjusted by BMI, age and sex. Abbreviations: CPR, C-protein reactive; IL-6, interleukin 6; IL-8, interleukin 8; IL-1b, interleukin 1 beta; IL-10, interleukin 10. Crosses represent control group; white squares represent prediabetes group and black circles represent T2D group

The proinflammatory molecules CRP, IL-6, IL-8 and IL-1b were positively correlated with serum SFRP4 concentrations (*r* = 0.305, 0.396, 0.406 and 0.353, respectively, *P* ≤ 0.001). Additionally, anti-inflammatory IL-10 was negatively correlated with serum SFRP4 concentrations (*r*=-0.3170, *P* < 0.001). As shown in Fig. 3, after adjustment for BMI, sex and age, the correlations remained between pro- and anti-inflammatory molecules and serum SFRP4 concentrations (CRP, *r* = 0.282; IL-6, *r* = 0.383; IL-8, *r* = 0.397; IL-1b, *r* = 0.350; and IL-10, *r*= -0.312). The p-values were < 0.001 for all correlations.

### Genotype analysis

To elucidate the contribution of SFRP4 genetics to metabolic factors, we carried out an analysis of allelic discrimination. In this analysis we included 264 T2D patients, to increase the statistical power from 37 to 48.5%. The allelic frequency of rs4720265 was 31.3% in the Mexican population, and the Hardy-Weinberg equilibrium was 0.962. An association with low HDL concentrations was identified in subjects with T2D and rs4720265 (Table 2, *P* < 0.05). Table 3 shows that in the control group, carriers of rs4720265 had lower levels of triglycerides, VLDL-C and IL-10 than noncarriers, independent of SFRP4 levels. Additionally, control subjects and carriers of rs4720265, had greater WC values than those of noncarriers. In addition, carriers of rs4720265 with T2D had lower HDL compared with noncarriers.

**Table 2:** Association analysis of rs4720265 with metabolic alterations in T2D subjects

SNP/Gen	Genotype	WC	Glucose mg/dL	Cholesterol mg/dL	Triglycerides mg/dL	HDL-C mg/dL	LDL-C mg/dL	Insulin µU/mL	HOMA-IR	HOMA-β
SFRP4 rs4720265	GG	99 ± 12	129 (102, 180)	192 ± 41	160 (117, 215)	46 (42, 57)	116 ± 34	9.1 (5.9, 14.2)	3.3 (1.7, 4.9)	40 (20, 80)
	GA/AA	99 ± 12	137 (113, 180)	190 ± 39	169 (130, 243)	46 (39, 55)	112 ± 36	9.1 (5.9, 13.6)	3.3 (2.0, 5.9)	39 (26, 73)
P		0.192	0.417	0.569	0.076	0.040	0.223	0.676	0.711	0.880

Abbreviations: WC, waist circumference; HDL-C, High density lipoprotein; LDL-C, Low density lipoprotein; HOMA-IR, Homeostatic Model Assessment for Insulin Resistance; HOMA-b, Homeostatic Model Assessment of beta cell function. In bold the mutant genotype is represented. Under the dominant model of inheritance, ANCOVA, in bold *P* < 0.05; fixed variables BMI, age and sex.

**Table 3:** Assessment of the rs4720265 effect in control and T2D subjects

		Control subjects		Prediabetes		T2D subjects	
		GG	GA/AA	GG	GA/AA	GG	GA/AA
N (M/F)		21 (7, 14)	23 (7, 16)	15 (9, 6)	22 (11, 11)	127 (50, 77)	137 (40, 97)
Age (years)		55.5 ± 16.1	54.4 ± 12.8	50.5 ± 11.1	53.6 ± 9.5	56.2 ± 10.9	54.6 ± 10.3
BMI (kg/m^2^)		28.2 ± 6.2	31.0 ± 5.4	33.1 ± 7.4	30.5 ± 3.7	29.7 ± 4.7	29.1 ± 5.3
WC (cm)	F	98 (86, 101) 95 (91, 97)	96 (88, 102) 103 (98, 104)^#^	116 (91, 128)	99 (96, 105)	97 (90, 103) 100 (91, 109)	98 (88, 104) 97 (90, 105)
	M			104 (95, 119)	103 (99, 107)		
WHR	F	0.90 (0.89, 0.93) 0.97 (0.95, 0.99)	0.91 (0.86, 0.96) 0.99 (0.98, 1.02)	0.95 (0.90, 0.98)	0.91 (0.89, 0.95)	0.92 (0.88, 0.96) 0.97 (0.94, 0.99)	0.91 (0.86, 0.95) 0.96 (0.95, 1.02)
	M			0.98 (0.92, 1.03)	0.98 (0.96, 1.00)		
SBP (mmHg)		120 (119, 140)	123 (115, 137)	134 (111, 143)	120 (118, 134)	130 (119, 146)	123 (115, 140)
DBP (mmHg)		80 (76, 80)	80 (75, 87)	84 (73, 90)	83 (72, 89)	80 (74, 90)	80 (73, 90)
Glucose (mg/dL)		88 (85, 95)	84 (80, 95)	96 (90, 107)	100 (94, 104)	128 (101, 178)	137 (113, 180)
HbA1c (%)		5.7 (5.5, 5.9)	5.6 (5.4, 5.7)	6.1 (5.9, 6.3)	6.1 (5.8, 6.3)	7.5 (6.5, 9.4)	7.6 (6.7, 9.0)
HOMA-IR		1.6 (0.5, 2.3)	1.4 (1.0, 2.7)	3.0 (1.8, 3.6)	2.7 (1.8, 3.9)	3.3 (1.7, 4.9)	3.3 (2.0, 5.9)
HOMA-b		85.4 (40.0, 135.4)	94.7 (70.3, 130.0)	98.7 (76.6, 178.4)	94.9 (75.4, 189.3)	39.6 (19.9, 79.6)	41.9 (27.1, 75.5)
Triglycerides (mg/dL)		170 (140, 184)	117 (93, 143)^#^	168 (149, 207)	144 (123, 234)	160 (117, 215)	169 (130, 243)
Total Cholesterol (mg/dL)		186 ± 38	180 ± 31	178 ± 27	183 ± 29	192 ± 41	190 ± 39
HDL-C (mg/dL)	F	47.6 ± 13.5 38.8 ± 8.9	48.8 ± 11.8 40.9 ± 10.8	41.6 ± 9.8	45.8 ± 8.0	54.8 ± 16.0 42.5 ± 9.1	49.1 ± 11.5^#^ 42.0 ± 9.8
	M			40.6 ± 3.9	40.4 ± 10.3		
LDL-C (mg/dL)		117 ± 41.8	117 ± 28.3	115 ± 29	123 ± 30	116 ± 34	112 ± 36
VLDL-C (mg/dL)		34 (28, 38)	23 (19, 29)^#^	36 (30, 42)	29 (25, 47)	45 (29, 55)	35 (28, 48)
SFRP4 (ng/mL)		157.2 (59.7, 183.9)	163.1 (80.6, 180.9)	178.5 (152.6, 198.8)	*157.0* (112.2, 197.5)	188.3 (169.0, 210.7)	192.9 (162.8, 213.0)
CPR (mg/L)		0.31 (0.08, 0.56)	0.41 (0.24, 0.67)	0.56 (0.43, 1.23)	0.26 (0.15, 0.39)^#^	0.46 (0.10, 0.80)	0.42 (0.19, 0.73)
IL-6 (pg/mL)		6.6 (4.6, 8.4)	6.4 (4.2, 7.1)	33.9 (30.5, 34.9)	30.6 (8.5, 36.6)	33.9 (31.8, 34.9)	32.9 (30.6, 34.9)
IL-1b (pg/mL)		5.7 (4.4, 6.4)	5.5 (4.5, 6.2)	32.2 (29.6, 34.5)	30.7 (6.9, 35.0)	32.2 (29.0, 34.1)	31.2 (27.6, 33.2)
IL-8 (pg/mL)		14.6 (11.1, 15.6)	14.1 (11.7, 15.8)	28.1 (25.2, 30.4)	27.3 (16.7, 30.6)	28.8 (26.4, 30.3)	29.6 (26.4, 30.9)
IL-10 (pg/mL)		63.7 (55.5, 65.2)	54.7 (54.0, 63.5)^#^	35.3 (33.5, 37.3)	34.6 (32.2, 53.5)	34.6 (33.1, 37.6)	35.3 (31.1, 37.1)

Data are presented as the means ± SDs or as medians (25th -75th percentiles). BMI, body mass index; WC, waist circumference; WHR, waist hip ratio; SBP, systolic blood pressure; DBP, diastolic blood pressure; HbA1c, glycated hemoglobin; HDL-C, high-density lipoprotein; LDL-C, low-density lipoprotein; VLDL-C, very low-density lipoprotein; BUN, blood urea nitrogen; AST, aspartate transaminase; ALT, alanine transaminase; SFRP4, secreted frizzled-related protein 4; CPR, C-protein reactive; Il-6, interleukin 6; IL-1b, interleukin 1 beta; IL-8, interleukin 8; IL-10, interleukin 10. In bold GG vs. GA/AA ^#^*P* < 0.05

## Discussion

At present, the increase in the consumption of foods with high contents of sugars and fats together with a lack of physical activity have considerably influenced the rates of T2D in Mexico. The importance and burden of T2D for the health system is constantly increasing, despite all efforts in primary prevention. Because it is a progressive disease that does not cause specific symptoms for many years, early diagnosis is of the utmost importance [22].

The present study demonstrated that patients with prediabetes and T2D have increased levels of SFRP4 protein in their plasma (Table 1; Fig. 1). This is in accordance with previous studies by Brix et al. [23], in which they showed that SFRP4 levels are elevated in patients with different types of diabetes (type 1 diabetes, T2D and latent autoimmune diabetes of the adult, LADA) compared with healthy controls. Additionally, Liu et al. [17] and Anand et al. [24] found the highest SFRP4 levels in T2D patients, followed by IGT and normal glucose tolerance groups. Furthermore, recent studies in patients with gestational diabetes demonstrated that increased SFRP4 levels in the first trimester of pregnancy were significantly associated with diabetes development and might be an important risk factor for this complication [25, 26].

We also observed that serum SFRP4 concentrations were positively correlated with BMI, WC, WHR, HbA1c, triglycerides and HOMA-IR (Fig. 2), although after adjustment for BMI, sex and age, the correlations with WC and WHR were lost. Some studies have shown that human subcutaneous obese adipose tissue has inadequate vascularization, hypoxia, and inflammation, which is proportional to insulin resistance [27, 28]. In the present study, 56% in the prediabetes group and 43% in the T2D group were obese. Garufi et al. [29] found that levels of SFRP4 correlated with body fat percentage, BMI, WHR and molecules of abdominal subcutaneous adipose tissue in lean and obese subjects that levels. Although in the present work the association of SFRP4 levels with WC or WHR was lost, it is important to mention that we did not measure visceral fat, which is a more accurate indicator of obesity than WC or WHR [30, 31]. Studies that consider different kinds of adipose tissues and different types of obesity are needed.

Madhi et al. [14] performed an analysis of global gene expression in human pancreatic islets. Among the genes that were overexpressed, *SRFP4* was found. In the same investigation, the increased levels of the protein SRFP4 were associated with reduced glucose tolerance due to decreased islet expression of channels of Ca^2+^, and as a consequence, the suppression of insulin exocytosis was observed. Hence, the increased levels of SFRP4 in T2D patients possibly can reduce insulin exocytosis due to a decrease in beta cell function (Table 1 shows values of HOMA-B), which causes an impaired glucose uptake by cells, and promotes a raise of blood glucose. This phenomenon could explain the significant positive correlation of serum levels of SFRP4 with HbA1c in the diabetes and prediabetes groups in the present study. This assertion is supported by the study of Taneera et al. [32], where the authors analyzed the expression of *SRFP4* in human islets from 63 donors. Among the gene co-expression networks and protein‒protein interactions studied, the expression of SFRP4 was strongly associated with an increase in HbA1c and a decrease in insulin secretion.

Furthermore, levels of SFRP4 were positively correlated with HOMA-IR, a commonly used method to estimate insulin resistance [33]. Some studies have shown a negative correlation between SFRP4 expression and insulin secretion, and plasma levels of SFRP4 have been positively correlated with insulin resistance and negatively correlated with insulin capacity to suppress lipolysis [14, 29]. Nunez Lopez et al. [34] measured circulating levels of cytokines and microRNAs (miRNAs) in lean and obese humans with prediabetes, with T2D and in healthy controls. Serum levels of SFRP4 were significantly and positively correlated with HOMA-IR and negatively correlated with the Quantitative insulin sensitivity check index (QUICKI) index. Additionally, Anand et al. [24] found a positive correlation between HOMA-IR and SFRP4 levels. Hence, the SFRP4 protein is related to insulin resistance as an impaired glucose metabolism.

Interestingly, the concentration of SFRP4 was correlated with high levels of proinflammatory molecules in the prediabetes and T2D groups (Fig. 3). This phenomenon supports the observation that interleukin-1β stimulates SFRP4 expression and therefore the existence of an inflammatory link between these factors [14]. Additionally, Taneera et al. [32] found that *SRFP4* expression was associated with the overexpression of inflammatory markers. In the study by Garufi et al. [29] mentioned above, circulating SFRP4 was directly proportional to abdominal subcutaneous adipose tissue inflammation, suggesting that SFRP4 inhibits abdominal subcutaneous adipose tissue vascularization, leading to tissue inflammation and ultimately insulin resistance in an endocrine manner, as suggested by the authors.

The increase in SFRP4 blood levels in people with prediabetes indicates that this protein has potential as an early biomarker of T2D and as a therapeutic target in pancreatic islet dysfunction, as suggested by Wilson et al. [35] However, further studies that consider the follow-up of prediabetes patients are required, and it would be interesting to include patients with complications, as well as studies that include insulin and glucagon measures, to understand the role of SFRP4 in T2D development.

On the other hand, to elucidate the contribution of SFRP4 as a genetic risk factor for metabolic disorders, we chose and identified the rs4720265 variant in participants based on the study by Boudin et al. [18] The authors reported that genetic variation in SFRP4 impacts on percentage body fat and height of Danish male population. Interestingly, rs4720265 noncarrier control subjects showed lipid disorders manifested by higher levels of triglycerides and VLDL-C (Table 2). Furthermore, noncarrier control subjects had higher levels of IL-10 than carriers. A recent study demonstrated that IL-10 is capable of producing an elevation of triglycerides as a modulator of lipoprotein levels [36]. Otherwise, rs4720265 male carriers had a higher WC than noncarriers. Also, we found a correlation of rs4720265 with low levels of HDL-C in carriers T2D subjects. There is only one study available that analyzes the association between genetic variants in SFRP4 and body composition in the Danish population; the authors identified six genetic variants associated with bone mineral density, percentage body fat and height, among which rs4720265 was found [18]. Hence, it is possible that rs4720265 is related to body composition and lipid homeostasis.

### Limitations of the study

One of the main limitations of the study is the relatively small sample size. This is a cross-sectional study and due to the distribution of patients attending at Hospital Juárez de México, most of them from across the country, it is difficult for the patients to return for their clinical follow-up. Hence, in a cohort study, we would be able to follow up the patients with prediabetes and observe the incidence of T2D to correlate the incidence with SFRP4 concentrations, and therefore evaluate SFRP4 as a prognostic factor for T2D. Thus, further longitudinal studies with larger sample size that can evaluate SFRP4 as a biomarker of T2D development are needed.

## Conclusions

Our results demonstrated that SFRP4 is an inflammatory mediator involved in T2D pathogenesis, and its levels could be increased even before T2D diagnosis. For the first time, we showed the genetic variant rs4720265 to correlate with low HDL concentrations in T2D subjects, suggesting a role in lipid balance. This association with HDL-C, along with the positive correlation of HOMA-IR with SFRP4 concentrations suggests an essential role of SFRP4 in developing of insulin resistance.

## Data Availability

The datasets used and/or analyzed during the current study are available from the corresponding author on reasonable request.

## Abbreviations

ALT: alanine transaminase
AST: aspartate transaminase
BMI: body mass index
BUN: blood urea nitrogen;
CRP: C Reactive Protein
DBP: diastolic blood pressure
HbA1c: glycated hemoglobin
HDL-C: high-density lipoprotein
HOMA-IR: homeostasis model assessment insulin resistance
IL-6: interleukin 6
IL-8: interleukin 8
IL-1b: interleukin 1 beta
IL-10: interleukin 10
LDL-C: low-density lipoprotein
SBP: systolic blood pressure
RSFRP4: secreted frizzled-related protein 4
T2D: type 2 diabetes
VLDL-C: very low-density lipoprotein
WC: waist circumference
WHR: waist hip ratio
